# Four Cysteine Residues Contribute to Homodimerization of Chicken Interleukin-2

**DOI:** 10.3390/ijms20225744

**Published:** 2019-11-15

**Authors:** Chen Deng, Hailiang Tan, Hongda Zhou, Mengyun Wang, Yan Lü, Jiacui Xu, Huanmin Zhang, Limei Han, Yongxing Ai

**Affiliations:** 1 College of Animal Science, Jilin University, 5333 XiAn Road, Changchun, Jilin 130062, Chinatanhl9915@163.com (H.T.); hongdazhou521@163.com (H.Z.); mengyun_wang1124@163.com (M.W.); lvyanty@jlu.edu.cn (Y.L.); jcxu@jlu.edu.cn (J.X.); 2 Key Laboratory of Zoonosis Research, Ministry of Education, College of Veterinary Medicine, Institute of Zoonosis, Jilin University, 5333 XiAn Road, Changchun, Jilin 130062, China; 3 Avian Disease and Oncology Laboratory, Agriculture Research Service, United States Department of Agriculture, 4279 East Mount Hope Road, East Lansing, MI 48823, USA; huanmin.zhang@usda.gov; 4 College of Animal Science and Veterinary Medicine, Shenyang Agricultural University, 120 Dongling Road, Shenyang, Liaoning 110866, China

**Keywords:** Interleukin-2, chicken, dimer, dissociation, disulphide bond, T cell

## Abstract

Interleukin-2 (IL-2) is a pleiotropic cytokine regulating the immune and nervous systems. Mammalian and bird IL-2s have different protein sequences, but perform similar functions. In the current study, two bands were detected by immunoblotting using an antibody against freshly purified chicken IL-2 (chIL-2). The molecular weight of the larger band was approximately twice as much of the chIL-2 monomer, although a chIL-2 complex or homodimer has never been reported. To explain this intriguing result, several dissociation reagents were used to examine the intermolecular forces between components of the proposed chIL-2 complex. It was found that intermolecular disulphide bond promotes homodimerization of chIL-2. Subsequently, mutation of Cys residues of chIL-2 revealed that mutation of all four Cys residues disrupted homodimerization, but a single, dual, or triple Cys mutation failed to disrupt homodimerization, suggesting that all four Cys residues on chIL-2 contribute to this dimerization. Functional analysis showed that both monomeric and dimeric chIL-2 consisting of either wild type or mutant chIL-2 were able to stimulate the expansion of CD4^+^ T cell in vivo or in vitro, and effectively bind to chIL-2 receptor. Overall, this study revealed that the recombinant chIL-2 purified from either *Escherichia coli* (*E. coli*) or *Spodoptera frugiperda* (Sf9) cells could homodimerize in vitro, with all four Cys residues on each chIL-2 protein contributing to this homodimerization, and dimerization and Cys mutation not impacting chIL-2 induced stimulation of chicken CD4^+^ T cells.

## 1. Introduction

Interleukin-2 (IL-2), a T cell growth factor, is primarily produced by CD4^+^ T cells [1,2] and plays pleiotropic roles in the immune system, such as in immune tolerance, response to microbial infection, and antitumor and antiviral effects [3,4]. IL-2 is an important factor for the regulation of proliferation, activation, differentiation, survival, and expansion of T cells [5,6]. IL-2 can significantly increase CD4^+^ T cell counts, and improve anti-viral immunity [7]. In contrast, IL-2 also can promote activation-induced cell death (AICD) of T cells by enhancing FasL expression [8]. Thus, IL-2 is one of pivotal regulator of immune homeostasis. IL-2 receptor, a heterotrimers consisting of IL-2Rα (CD25), IL-2Rβ (CD122), and γc (CD132) subunits, is indispensable to bioactivity of IL-2. IL-2 receptor shares a common β and γ subunit with the IL-15 receptor, but the private α subunit [9]. CD25 is a transmembrane glycoprotein with a molecular weight of 55 kDa, and expressed by most T, B, and NK cells only post antigen or IL-2 activation [10]. CD25 specifically binds to IL-2 and enhances receptor trimer affinity, resulting in the drastic promotion of T cell proliferation [9]. As a regulatory T cell subpopulation, CD4^+^CD25^+^ T cells play an important role during virus infection [10,11]. The brain nerve cells also contain an IL-2 receptor, with IL-2 being able to penetrate the blood-brain barrier and participating in the regulation of the brain and nervous system [12,13]. IL-2 encoded by different species performs the same function, while having a different amino acid sequence. For example, chicken IL-2 (chIL-2) contains four Cys residues, while human IL-2 (hIL-2) has three. It is a secretory protein, expressed as a precursor containing an N-terminal signal peptide, which matures into the functional IL-2 cytokine following cleavage of the signalling sequence in the cell [2,14]. The mature mammalian IL-2 is able to stimulate bird splenic T cells, while bird IL-2 is not able to stimulate mammalian T cells. In vitro, mature IL-2 is usually applied as a stimulant in combination with concanavalin A (ConA) or phytohemagglutinin (PHA) to induce the expansion of T cells in cell culture. Human IL-2 has also been approved as a drug for the treatment of metastatic renal cell carcinoma (metastatic RCC) in some countries [15]. The mature hIL-2 can induce CD4^+^ T cell expansion in HIV-infected patients [16]. Exogenously expressed mature chIL-2 can increase the immune response against Marek’s disease virus (MDV) [17]. ChIL-2 was found to be a T cell growth factor and was able to maintain T cells continually in long-term culture [18], and participates the replication and maturation of T cells [19]. ChIL-2 has been used as a vaccine adjuvant to improve the vaccines response to *Eimeria* parasites and poultry virus [20,21]. The various infectious diseases of poultry are a major threat to the poultry industry and human health. Although the use of antibiotics is conducive to the disease resistance and growth of poultry, antibiotics were beginning to be banned from feed [22]. Thus, improving the immunity of poultry is an environmentally friendly strategy to defend against some infectious diseases [23]. As a vaccine adjuvant, the chIL-2 has attracted much more attention. The revelation of properties, functions, and structural characteristics of chIL-2 are the key to safe and rational use of chIL-2. When we investigated the use of chIL-2 as an immunoregulator for the defence of chicken Marek’s disease (MD), that is caused by the specific infection and transformation of MDV on chicken immune cells, especially CD4^+^ T cells, the potential oligomerization of chIL-2 was probed during the purification and administration of chIL-2. To reveal the possible oligomerization mechanisms in the current study, various dissociation reagents and mutational analysis were applied. Chicken CD4^+^ T cells were applied to analyse whether these mutants and dimeric chIL-2 differed from wild type and monomeric chIL-2 in stimulation function in vitro or in vivo. CD25 was used as a marker to characterize the combination between chIL-2 monomer/dimer and chIL-2R.

## 2. Results

### 2.1. Purified ChIL-2 Formed a Dimer

**Figure 1:**
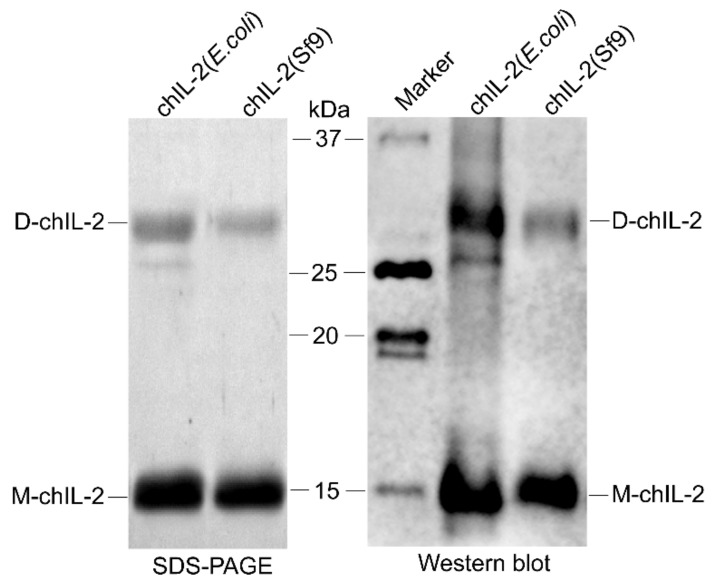
Identification of purified chIL-2. Purified chIL-2 proteins were identified by SDS-PAGE and Western blotting. D-chIL-2, chIL-2 dimer; M-chIL-2, chIL-2 monomer; chIL-2 (*E. coli*), chIL-2 protein purified from *E. coli* cells; chIL-2 (Sf9), chIL-2 protein purified from Sf9 cells; Marker, protein standard marker.

In Figure 1, SDS-PAGE shows that hexa-His tagged chIL-2 purified from Sf9 cells or BL21(DE3) cells presented two bands at approximately 15 and 30 kDa each, detected by an antibody against chIL-2.

### 2.2. Increasing Temperature Promotes Dimerization of ChIL-2

**Figure 2:**
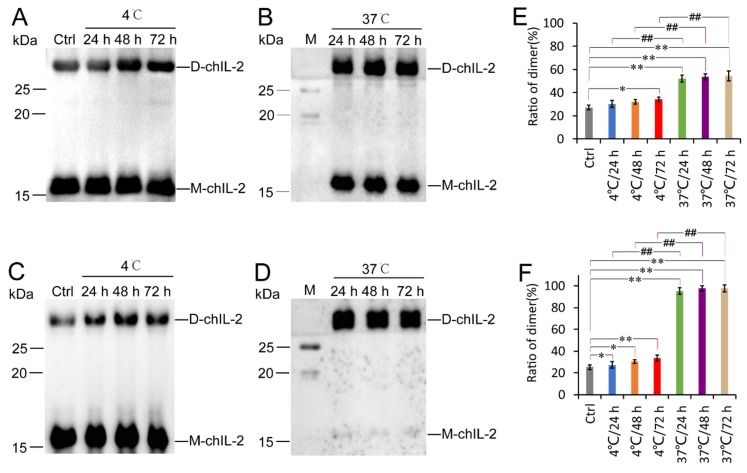
Effect of temperature on chIL-2 dimerization. (**A**), (**B**), (**C**) and (**D**) are western blot images of the chIL-2 monomer and dimer using an antibody against chIL-2. ChIL-2 purified from *E. coli* cells was incubated at 4 °C (**A**) or 37 °C (**B**) for different durations. Purified chIL-2 from Sf9 cells was incubated at 4 °C (**C**) or 37 °C (**D**) for different durations. Quantification of chIL-2 dimers in (**A**), (**B**), (**C**), and (**D**) was shown in (**E**) for *E. coli* cells and (**F**) for sf9 cells. The plotted data represent mean values ± standard deviation, and are the average of three independent experiments. Ctrl, control group of purified chIL-2 that was not incubated; M, in (**B**) and (**D**), protein standard marker; D-chIL-2, dimeric chIL-2; M-chIL-2, monomeric chIL-2. *, *p* < 0.05, **, *p* < 0.01 compared to the control group. ##, *p* < 0.01 compared 4 °C group to 37 °C group at the same incubation time.

The effect of temperature on chIL-2 dimerization was tested by incubating purified chIL-2 at 4 °C and 37 °C for 24, 48, and 72 h. As shown in Figure 2, chIL-2 purified from either *E. coli* or Sf9 cells were up to approximately 34% homodimeric, when incubated at 4 °C. ChIL-2 from *E. coli* cells dimerized up to about 55% when incubated at 37 °C, whereas chIL-2 purified from Sf9 cells formed more than 98% dimer. These results suggest that increasing incubation temperature enhanced the homodimerization of chIL-2 purified from either *E. coli* or Sf9 cells. Prolonging incubation time beyond 24 h did not significantly increase the rate of dimerization of chIL-2. Different expression sources of chIL-2 demonstrated different dimerization ability.

### 2.3. Reducing Agents Dissociated ChIL-2 Homodimer

**Figure 3:**
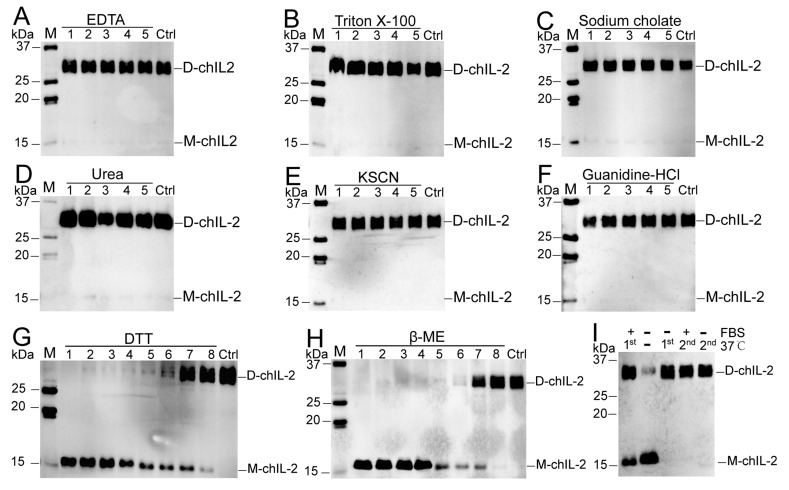
Effect of some dissociation reagents on chIL-2 dimer. M, protein standard marker; Ctrl, chIL-2 without treatment with dissociation reagents. Labels 1~5 in (**A**–**F**), five-fold serial dilution of dissociation reagents. 1~8 in (**G**–**H**), eight-fold serial dilutions of reducing agents. D-chIL-2, dimeric chIL-2. M-chIL-2, monomeric chIL-2. In (**I**), +, incubation with FBS; −, absence of FBS or incubation at 37 °C. 1st, the first incubation of purified chIL-2 at 37 °C; 2nd, the incubation within FBS post the 1st incubation without FBS at 37 °C.

**Figure 4:**
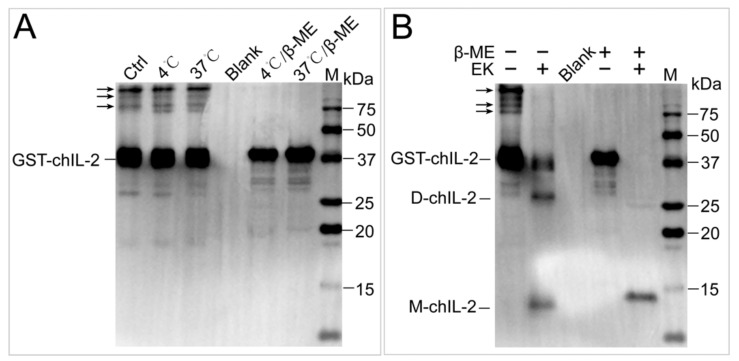
The effect of GST tag on dimerization of chIL-2. (**A**), the effect of temperature and β-ME on GST-chIL-2 dimerization. (**B**), the effect of β-ME on the dimerization of chIL-2 generated by cleavage of GST tag. EK, enterokinase. M, protein standard marker. Ctrl, chIL-2 without temperature and β-ME treatment. Blank, 1× loading buffer without reducing agent and chIL-2 protein.

Figure 3A–F show the inability of EDTA, Triton X-100, sodium cholate, urea, NaSCN and guanidine hydrochloride to dissociate the chIL-2 dimer, while demonstrating the ability of DTT and β-ME (Figure 3G,H) to dissociate chIL-2 dimer to monomer in a concentration-dependent manner. This result implies that chIL-2 dimerization primarily involves disulphide bonds. As shown in Figure 3I, monomeric chIL-2 incubated with FBS does not fully dimerize, but FBS did not dissociate dimeric chIL-2 either. This result suggests that chIL-2 could be stable in the serum during the in vivo experiment or in vitro cell culture, although its dimerization is subject to some degree of interference in the presence of serum. GST tagged chIL-2 was able to dimerize and oligomerize (Figure 4), but in very minute quantities. Untagged chIL-2 generated from EK cleavage of GST-chIL-2 was unable to dimerize with reducing agents disrupting this dimerization. This result confirmed that chIL-2 dimerized through disulphide bond formation. Although oligomeric forms of GST-chIL-2 were detected, oligomers of untagged or hexa-His tagged chIL-2 were hardly detected, suggesting that steric hindrance of chIL-2 may further block oligomerization of chIL-2 dimer. This observation was verified by an in silico SWISS model, which showed that the four Cys residues forming the disulphide bonds were located on the same region of the chIL-2 surface (Figure S1).

### 2.4. The ChIL-2 Mutant with Four Cys Residue Mutations Fails to Form Dimeric ChIL-2

**Figure 5:**
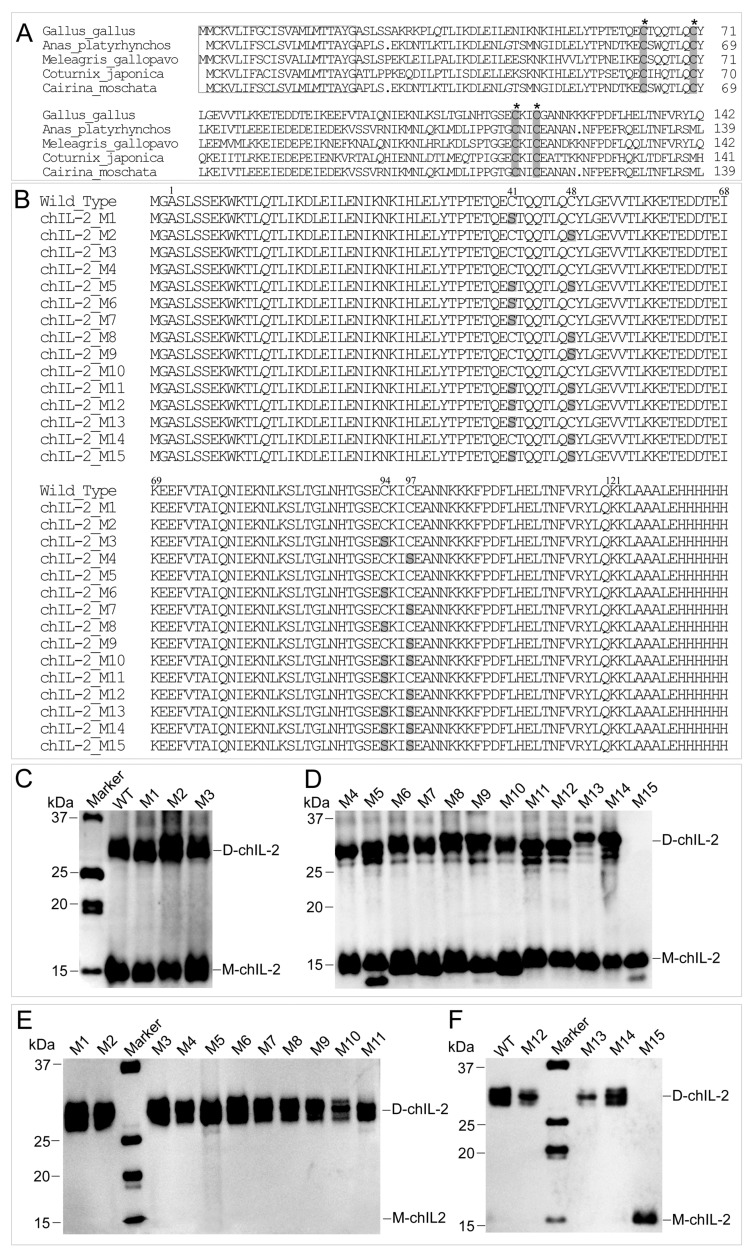
Determination of cysteine sites involved in dimerization. (**A**), the alignment of avian IL-2 precursors. The sequences in rectangles are the signal peptide sequences, while other sequences are that of the mature IL-2. The residues highlighted in grey and indicated by an asterisk (*) are the conserved Cys residues. Dot indicates no amino acid. (**B**), the alignment of chIL-2 wild type and mutants. The numbers on the top of sequence indicate the residue position of mature chIL-2 protein from Ala^1^ to Lys^121^. Other residues were generated from expression vector during chIL-2 expression. The residues highlighted in grey are Ser residues mutated from Cys. (**C**) and (**D**), western blotting of chIL-2 purified from *E. coli*. (**E**) and (**F**), chIL-2 purified from Sf9. M1~M15, chIL-2 mutants with different Cys mutation combinations (refer to Figure 5B and Table 1). Marker, protein standard marker. WT, wild type chIL-2.

Based on the results from the reducing agent assay, amino acid sequences of different IL-2s were aligned, which showed that four Cys residues are highly conserved in bird IL-2 (Figure 5A), while mammalian IL-2 contains three conserved Cys residues (Figure S2). Further, chIL-2 shares no identity with human IL-2 (Figure S3). Based on chIL-2 protein sequence, the mutants containing various combinations of Cys residue(s) mutation(s) were generated to identify which Cys residue contributes to chIL-2 dimerization (Figure 5B). In chIL-2, mutants purified from *E. coli* and Sf9 cells, only the chIL-2 mutant containing all four conserved Cys residue mutations was not able to form dimeric chIL-2, post incubation (Figure 5C–F). This result suggests that every Cys residue in chIL-2 contributes equally to homodimerization.

### 2.5. Both Dimeric and Monomeric ChIL-2 Stimulated T Cells Expansion

**Figure 6:**
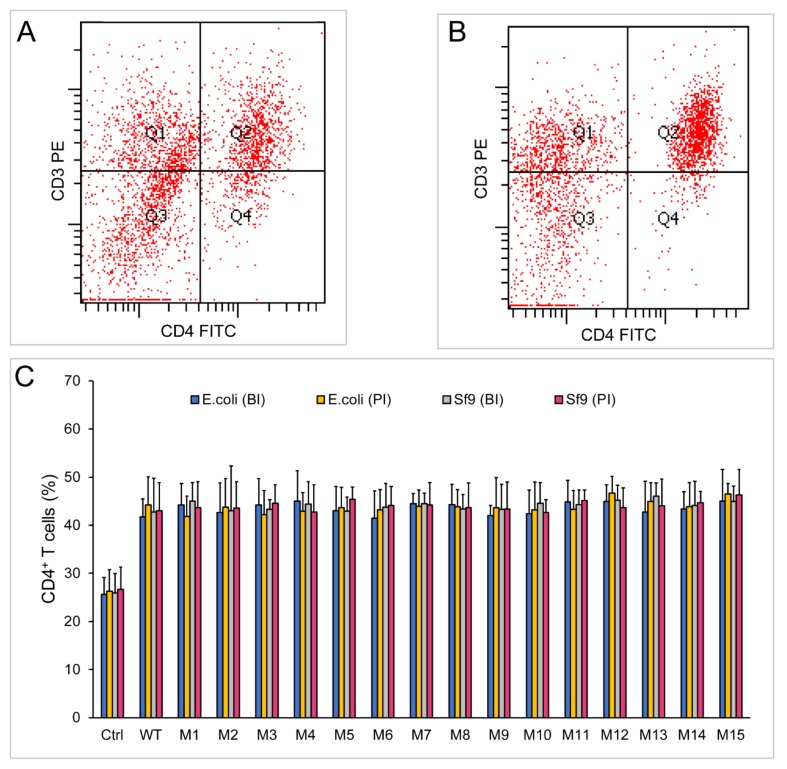
Stimulation assay of chIL-2 monomer and dimer in vitro. (**A**) and (**B**), immunofluorescence analysis of CD4^+^ T cells by flow cytometry using PE-conjugated anti-CD3 (CD3 PE) and FITC-conjugated anti-CD4 (CD4 FITC) antibodies before (**A**) or post (**B**) stimulation of chIL-2. (**C**), the bar graph of CD4^+^ T cell proportions. Before stimulation of T lymphocytes, each source of chIL-2 proteins was divided into two groups, one group was unincubated at 37 °C (before incubation, BI), and the other group was treated with chIL-2 dimer generated from 37 °C incubation (post incubation, PI). The plotted data represent mean values ± standard deviation, and are the average of three independent experiments.

**Figure 7:**
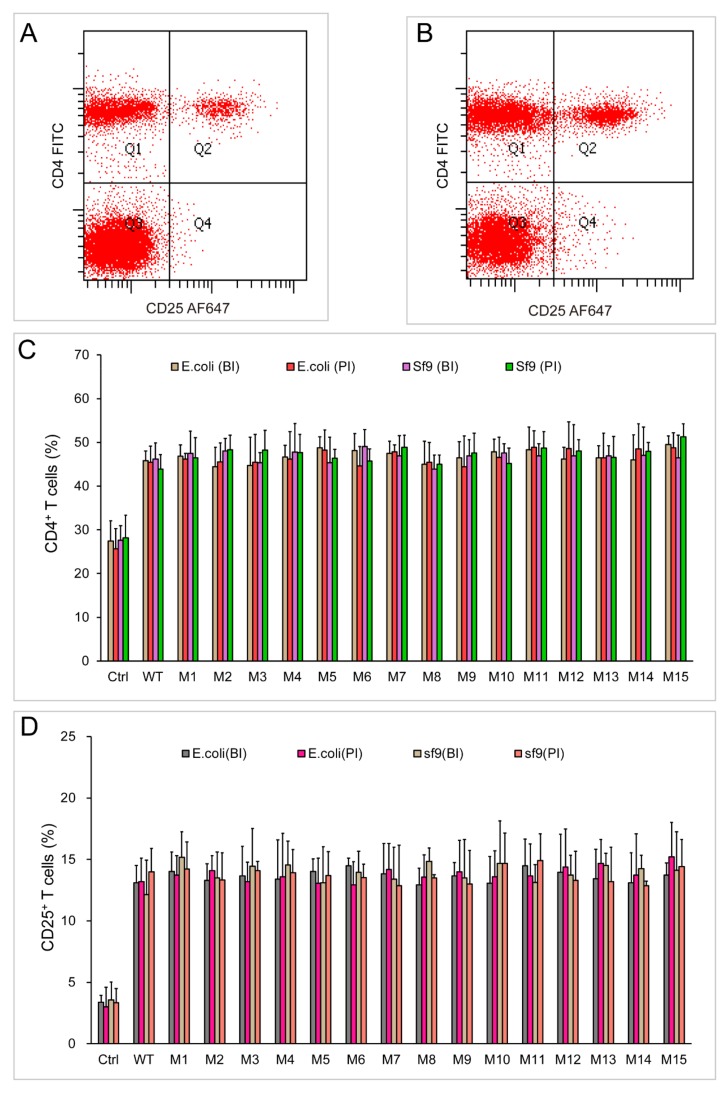
Stimulation assay of chIL-2 monomer and dimer in vivo. (**A**) and (**B**), immunofluorescence analysis of CD4^+^ and CD25^+^ T cells by flow cytometry using FITC-conjugated anti-CD4 (CD4 FITC) and Alexa Fluor 647-conjugated anti-CD25 (CD25 AF647) antibodies before (**A**) or post (**B**) stimulation of chIL-2. (**C**), the bar graph of CD4^+^ T cell proportions; (**D**), the bar graph of CD25^+^ T cell proportions; BI, chIL-2 protein was not treated with incubation at 37 °C; PI, chIL-2 was treated with incubation at 37 °C. The plotted data represent mean values ± standard deviation, and are the average of three independent experiments.

The stimulation functions of monomeric or dimeric chIL-2 were further tested on chicken splenic T cells either in vivo or in vitro. Compared with the control group, both monomer and dimer of chIL-2 could significantly increase the proportion of CD4^+^ or CD25^+^ T cells (*p* < 0.01) in vitro (Figure 6) or in vivo (Figure 7). The ability of chIL-2 dimer to stimulate T cell expansion was not significantly different from that of monomeric chIL-2. The ability of chIL-2 mutants to stimulate T cell expansion was not significantly different from that of wild type chIL-2. This suggests that Cys mutation(s) did not impact stimulation function of chIL-2, and dimeric chIL-2 still remain the same stimulation function as monomeric chIL-2, but no significant difference was found.

### 2.6. Different Forms of ChIL-2 can Effectively Bind to ChIL-2 Receptor

**Figure 8:**
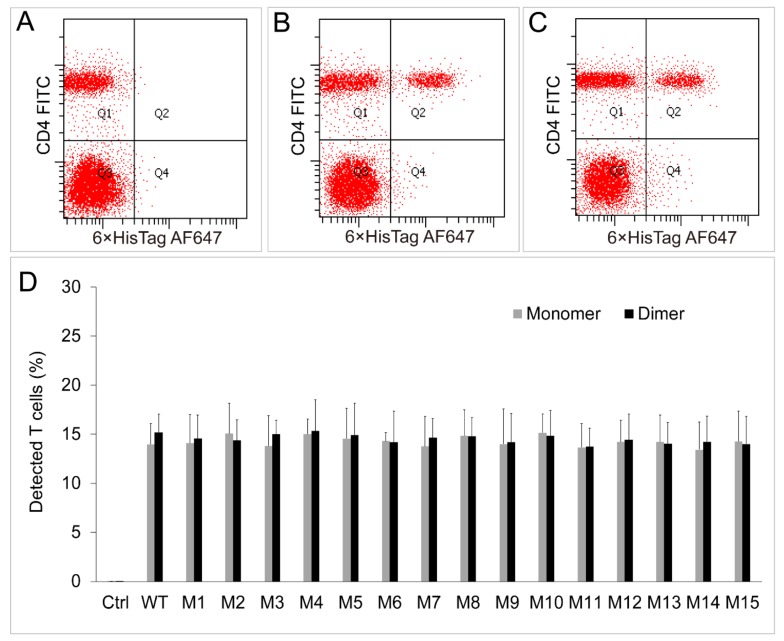
Binding assays between chIL-2 monomer/dimer and chIL-2R in vivo. (**A**), (**B**) and (**C**) were immunofluorescence analyses of the T cells bound hexa-His tagged monomeric or dimeric chIL-2 by flow cytometry using FITC-conjugated anti-CD4 (CD4 FITC) and Alexa Fluor 647-conjugated anti-hexa-His (6×HisTag AF647) antibodies before (**A**) or post stimulation of monomeric (**B**) or dimeric (**C**) chIL-2. The proportions of detected T cells with each antibody were plotted as a bar graph (**D**). Ctrl, the proportions of T cells without the stimulation of any form of chIL-2; Monomer, the proportions of T cells that stimulated by sf9 source of monomeric chIL-2 protein; Dimer, the proportions of T cells that stimulated by sf9 source of dimeric chIL-2 protein. The plotted data represent mean values ± standard deviation, and are the average of three independent experiments.

**Figure 9:**
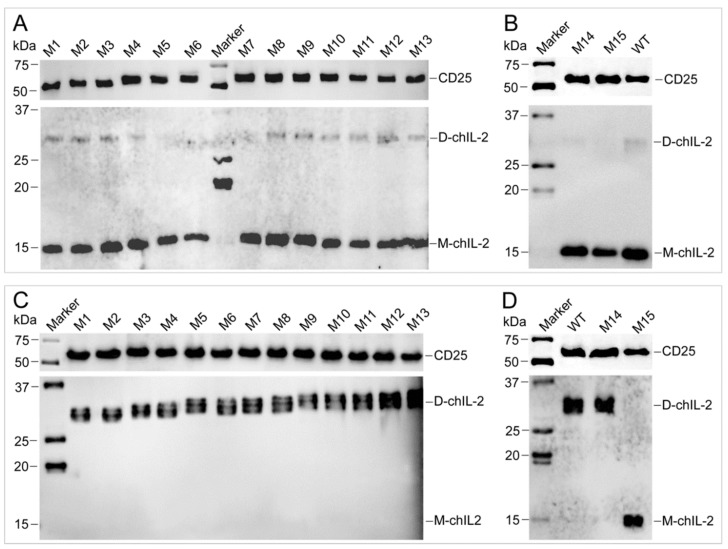
Pull-down assay for characterization of the interaction between chIL-2 and CD25 in vivo. Hexa-His tagged chIL-2 was purified from the T cells that were isolated from hexa-His tagged chIL-2 treated chicken. Monomeric (lower panel in **A** and **B**) or dimeric (lower panel in **C** and **D**) chIL-2 were detected by anti-chIL-2 antibody, respectively. CD25 protein was pulled down along with monomeric or dimeric chIL-2 purification, and detected by anti-CD25 antibody (upper panel in **A**, **B**, **C** and **D**). D-chIL-2 and M-chIL-2 are the recombinant chIL-2 monomer and dimer purified from Sf9, respectively. M1~M15, chIL-2 mutants with different Cys mutation combinations (refer to Figure 5B and Table 1). Marker, protein standard marker. WT, wild type chIL-2.

The binding assays by flow cytometry and western blotting were carried out to reveal whether different forms of chIL-2 can effectively bind to chIL-2 receptor. The flow cytometry results showed that no His tag was detected on T cells in the control group (Figure 8A) while hexa-His tagged chIL-2 was detected in the chIL-2 monomer (Figure 8B) and dimer (Figure 8C) treated groups. The amount of chIL-2 monomer bound on T cells was not significantly different from that of chIL-2 dimer, and no significant difference was found between wild type and mutant groups (Figure 8D) as well. The pull-down assays showed that CD25 could be pulled down along with monomeric or dimeric chIL-2 purification from the T cells that were isolated from chIL-2 treated birds. This result further confirmed that different forms of chIL-2 prepared in the current study can effectively bind to chIL-2 receptor (Figure 9).

## 3. Discussion

Dynamic protein-protein interactions regulate intra- and extra-cellular activities of proteins. These interactions between protein molecules may induce the formation of protein complexes, such as the formation of homo- or hetero- dimers or higher oligomerization [24]. Each oligomeric form may present distinctive functional roles in comparison to other oligomeric forms. Protein-protein complexation allows limited genes within the genome to play unlimited functions, with expanded protein function and complexity [24]. Dimerization, including homodimerization and heterodimerization of proteins, is a very common phenomenon in the biological system. It is an important regulatory mechanism of activity of proteins, such as in enzymes, receptors, and ion channels. Proteins usually form complexes through non-covalent bonds, such as hydrogen bonds, ionic bonds, van der Waals interactions, and hydrophobic bonds [25]. Some proteins also dimerize through disulphide bonds, such as the homodimerization of NEMO proteins [26] and the heterodimerization of TWIK-1/TREK-1 proteins [27]. It has been reported that murine IL-2 (mIL-2) could homodimerize through disulphide bonds via one Cys residue Cys140, out of three Cys residues [28], whereas hIL-2 homodimerizes through an intramolecular disulphide bond between Cys58 and Cys105 residues [29]. However, chIL-2 has a sequence distinct from that of mammalian IL-2, and hence, the mechanism of chIL-2 homodimerization cannot be directly inferred from the mechanisms of dimerization in mammalian, human, or mouse IL-2, with highly conserved Cys residues forming disulphide bonds in different ways. Thus, we tested various dissociation reagents to analyse the intermolecular forces involved in dimerization of chIL-2. Mutation analysis revealed that all four Cys residues participate in disulphide bond formation, which is different from the case of mIL-2 and hIL-2. Our results reveal that chIL-2 dimerizes through disulphide bonds rather than other intermolecular forces, and four Cys residues contribute to disulphide bond formation.

Upon the discovery that chIL-2 dimerizes through disulphide bonds, with no further oligomerization, we attempted to explore the distribution of four Cys residues on chIL-2 through the structural analysis of chIL-2 by X-ray crystallography or nuclear magnetic resonance spectroscopy (NMR). However, this attempt failed likely because of the heterogeneity in the sample containing a mixture of chIL-2 monomers and various dimers. Therefore, we applied an in silico approach and used the SWISS-MODEL to predict the chIL-2 structure. The model showed that all four Cys residues were centrally distributed in the same area of the chIL-2 surface. This is consistent with the result that chIL-2 cannot form higher oligomers. The probable reason is that the two chIL-2 monomers forming the dimer sterically hinder the entry of a third chIL-2 monomer, thereby inhibiting the formation of a third disulphide bond and restraining further oligomerization. In addition, although mass spectrometry (MS) is a common method for determining the position of disulphide bonds [30], for chIL-2, which forms a homodimer, site-directed mutagenesis is a better way to determine the position of intermolecular disulphide bonds. Our current study eventually determined that all four Cys residues participate in disulphide bond formation.

ChIL-2 expressed in bacterial or Sf9 cells demonstrate different homodimerization efficiencies. ChIL-2 expressed in eukaryotic cells can completely dimerize, while the chIL-2 expressed in prokaryotic cells homodimerizes partially, indicating that protein folding or post-translation modifications may also be important factors in the formation of chIL-2 dimer. We did not find any impact of glycosylation on chIL-2 dimerization through secretary expression of chIL-2 fused to the Honeybee melittin signalling sequence (HMS) in Sf9 cells (data not shown). In any case, there is indeed a significant difference in the formation of dimers between eukaryotic-derived and prokaryotic-derived chIL-2. As for the effect of temperature, 4 °C is the temperature at which chIL-2 protein is purified, and its dimerization was found. The common temperature for cell culture or physiological temperature is 37 °C. The temperature 40 °C was used to mimic the bird body temperature in preliminary work, but no difference was found between 40 °C and 37 °C groups (data not shown). Two common temperatures, 4 °C and 37 °C, are different greatly, which can directly reflect the influence of temperature on the formation of chIL-2 dimer. Therefore, these two temperatures were chosen to test the influence of temperature on chIL-2 dimerization.

IL-2, as a pleiotropic cytokine, participates in various immune processes and plays essential roles in immunoregulation [31]. It has been reported that mouse IL-2 is retained in blood vessels by heparan sulphate and released as a dimer from vessel tissue by heparinase [32]. Dimeric IL-2 is also highly cytotoxic to cells expressing the IL-2 receptor [33]. Human and murine IL-2 receptors bind to the homodimeric form of the ligand [28,34]. The linear dimer of IL-2 generated by genetic engineering retains the activity of IL-2 monomer [35]. Since IL-2 can stimulate CD4^+^ T cell expansion or enhance the effect of vaccine against the viruses that specifically infect CD4^+^ T cells [16,17,19], the stimulation assays of monomeric and dimeric chIL-2 on CD4^+^ T cells were carried out to reveal the functional difference between two forms of chIL-2. The current study shows that there is no significant difference in stimulation of chicken splenic CD4^+^ and CD25^+^ T cells between monomeric and dimeric chIL-2, and both forms of chIL-2 were able to effectively bind to chIL-2 receptor alpha subunit (CD25), mutants still can bind to chIL-2R and stimulate T cells expansion. Combining with the reports in mammalian IL-2, it could be inferred that natural chIL-2 homodimer probably present in chicken blood or tissue, and this dimeric chIL-2 may function in a certain tissue rather than on T cell, although it has not been isolated or detected due to trace amount. Further research is needed to isolate natural chIL-2 dimer or use recombinant dimeric chIL-2 to characterize the interaction with other types of tissues, and reveals its physiological function and whether dimeric chIL-2 is also cytotoxic to other immunocytes or neurocytes, similar to mammalian IL-2. More detailed functional studies of homodimeric chIL-2 in vivo will improve the rational application of chIL-2 as a drug in clinical practice.

## 4. Materials and Methods

### 4.1. Expression and Purification of ChIL-2

*ChIL-2* gene encoding mature chIL-2 protein (amino acid 23–143) without the N-terminal signalling sequence (1–22) was amplified by PCR from the full length chIL-2 precursor (NP_989484.1) donated by Xicheng Zhang (Jilin University, Changchun, China) [36]. *ChIL-2* gene was subcloned into a pET28a (Merck, Temecula, CA, USA) expression vector with NcoI/HindIII restriction enzyme sites and fused to a C-terminus hexa-His-tag using primers P1 and P2 (Table 2). The gene was subcloned into a pFASTBac-1 donor plasmid (Thermo Fisher Scientific Co., Waltham, MA, USA) with SalI/NotI restriction enzyme sites using primers P3 and P4 (Table 2) and fused a C-terminus hexa-His-tag. The GST tag gene in pGEX4T3 plasmid (GE Healthcare, Waukesha, WI, USA) and the *chIL-2* gene were amplified, respectively, with two pairs of primers, P5/P6, P7/P8, and then subcloned into the pFASTBac-1 donor plasmid, allowing GST tag to fuse to the chIL-2 N-terminus, with an enterokinase (EK) cleavage site introduced between GST and chIL-2. These constructs were verified by sequencing.

For expression and purification of chIL-2 in *E. coli*, pET-28a-chIL-2-His6 recombinant expression plasmid was transformed into BL21(DE3) competent cells. Recombinant expression of chIL-2 in BL21(DE3) cells was induced by 0.2 mM isopropyl β-d-1-thiogalactopyranoside (IPTG) for 12 h at 16 °C followed by centrifugation to harvest the cells. The cell pellet was resuspended in lysis buffer (50 mM Tris·HCl, pH 7.4, 500 mM NaCl, 10 mM imidazole, 10% (*v*/*v*) glycerol, Protease Inhibitor Cocktail (MilliporeSigma Co., St. Louis, MO, USA)) followed by sonication to lyse the cells. The cell lysate was clarified by centrifugation (12,000× *g*, 1 h, 4 °C) and filtration (0.22 μm). The supernatant was mixed with Ni-NTA agarose (Thermo Fisher Scientific Co.), which was washed and pre-equilibrated with lysis buffer, for binding for 1 h at 4 °C. The resin bound protein was washed extensively with lysis buffer and eluted with elution buffer (50 mM Tris·HCl, pH 7.4, 500 mM NaCl, 10% (*v*/*v*) glycerol) containing a gradient imidazole concentration of 30, 80, 120 and 150 mM. The purity of the eluted fractions was determined by SDS-PAGE. The pure fractions were pooled together and dialyzed in dialysis buffer (10 mM Na_2_HPO_4_, 2 mM KH_2_PO_4_, 137 mM NaCl, 2.7 mM KCl, pH 7.4, 10% glycerol), followed by filter sterilization. Aliquots of the purified protein were either stored in liquid nitrogen or directly used.

For expression and purification of chIL-2 in Bac-to-Bac system (Thermo Fisher Scientific Co.), the recombinant donor plasmids pFASTBac-chIL-2-His6 or pFASTBac-GST-chIL-2 were transformed into DH10BAC competent cells. According to the manual, white colonies containing recombinant bacmids were selected with three times plating and recombinant bacmids were extracted with E.Z.N.A. Endo-Free BAC/PAC DNA kit (OMEGA Bio-tek, Norcross, GA, USA). Recombination of the *chIL-2* gene was confirmed by PCR. Endotoxin-free recombinant chIL-2 bacmids were transfected into Sf9 cells with X-treme GENE HP DNA Transfection Reagent (Roche, Mannheim, Germany) for recombinant baculovirus generation. The large-scale Sf9 cells were infected with chIL-2 recombinant baculovirus with the multiplicity of infection (MOI) = 1 for 3 days, and then collected and lysed for purification of hexa-His tagged chIL-2 on Ni-NTA Agarose column (Thermo Fisher Scientific Co.) or GST tagged chIL-2 on Glutathione agarose column (Thermo Fisher Scientific Co.). Enterokinase (Thermo Fisher Scientific Co.) was used for the excision of GST tag from purified GST-chIL-2 under the recommended reaction conditions.

### 4.2. Homodimerization of ChIL-2 Protein

Hexa-His tagged or GST tagged chIL-2, which was freshly purified from *E. coli* or Sf9 cells was treated with 5× protein loading buffer without reducing reagent (125 mM Tris·HCl, pH 6.8, 5% (*w*/*v*) SDS, 0.2% (*w*/*v*) bromophenol blue, 25% (*v*/*v*) glycerol) and boiled. SDS-PAGE (15% gel percentage) was performed to identify purified chIL-2 followed by western blotting using mouse anti-chIL-2 IgG antibody (1:5000 dilution, Bio-rad, Hercules, CA, USA). The GST tag was detected using the mouse anti-GST antibody (1:5000, Abcam, Cambridge, MA, USA). The secondary antibody used was goat anti-mouse IgG conjugated to horseradish peroxidase (HRP) (1:5000, Abcam). The band position detected by anti-chIL-2 antibody was calculated using ImageJ software (V1.52p, NIH, Bethesda, MD, USA) [37].

### 4.3. The Effect of Temperature on ChIL-2 Dimerization

Purified chIL-2 was incubated in phosphate buffered saline (PBS) buffer at 4 °C and 37 °C for 24, 48 and 72 h, respectively, to evaluate the effect of temperature on chIL-2 dimerization. Western blot analysis was performed for the detection of chIL-2 dimer following the same protocol as above. The ratio of monomeric and dimeric chIL-2 were detected by grey analysis using ImageJ software [37]. One-way ANOVA in SPSS 25 (IBM Corp., Armonk, NY, USA) was used to analyse the significance of the differences between non-incubated and incubated group, or between different incubation temperature treatments at the same incubation time. There were three repetitions for each treatment point (*n* = 3).

### 4.4. Dissociation of the ChIL-2 Dimer

Sf9-derived chIL-2 was incubated at 37 °C for 24 h to generate the chIL-2 dimer (complete dimer). Equal amounts of the chIL-2 dimer were incubated in PBS buffer containing dissociation reagents in a series of five-fold dilutions from the initial concentrations of 4 M KSCN [38], 10% sodium cholate [39], 20% Triton X-100 [40], 8 M urea, 4.8 M guanidine hydrochloride [41], 215 mM EDTA [42], 6 M β-ME and 0.8 M DTT, at 37 °C for 1 h. To test the effect of foetal bovine serum (FBS) on dimerization of chIL-2, freshly purified chIL-2 (mostly monomeric) and chIL-2 dimer (post incubation at 37 °C for 24 h) were added to the T cell culture medium (RPMI 1640 medium supplemented with 10% FBS (Gibco, Waltham, MA, USA), 100 U penicillin, and 100 mg streptomycin (Gibco)) and incubated at 37 °C for 24 h. Western blot was employed for the detection of chIL-2. ChIL-2 not incubated at 37 °C or FBS were used as controls.

### 4.5. Determination of Cysteine Sites Involved in Dimerization

Site-directed mutagenesis was performed to generate single, double, triple or quadruple mutants of the chIL-2 protein residues Cys41, Cys48, Cys94, and Cys97 using the *pfu* turbo DNA polymerase (Agilent Technologies, CA, USA) and mutation primers (Table 3), in accordance with the instruction manual. Fifteen mutants were generated using different combinations of Cys mutations (Table 1) and the mutants were expressed using the pET28a-chIL-2 and pFASTBac-chIL-2-His6 plasmids in BL21(DE3) *E. coli* cells and Sf9 cells, respectively. The mutants were purified by binding on a Ni-NTA agarose column. Equal amounts of purified chIL-2 mutant or wild type proteins were incubated in PBS buffer at 37 °C for 24 h. Western blot was employed to identify the dimer or monomer of wild type or mutated chIL-2.

### 4.6. Identification of Differences in Stimulatory Activity between Monomeric and Dimeric ChIL2

To investigate whether the stimulation function of dimeric chIL-2 differs from that of monomeric chIL-2, chicken T cells were isolated to test the expansion of CD4^+^ T cell subset. Briefly, 4-week old specific pathogen-free (SPF) White Leghorn chickens (Boehringer Ingelheim Vital Biotechnology Co. Ltd., Beijing, China) were used for splenic T cell isolation [43]. T cell isolation was performed in accordance with the instruction manual of the chicken spleen lymphocyte separation kit (TBD Science, Tianjin, China). A nylon wool column (Polysciences, Inc. Warrington, PA, USA) was used to remove adherent cells and isolate T cells [44]. Based on a previous report [19], T cells were induced with ConA to a final concentration of 4 μg/mL for 20 h in T cell culture medium at 37 °C, followed by inhibition by addition of 0.1 M Methyl α-d- mannopyranoside (α-MM) (MilliporeSigma Co.) for 30 min. T cells were then washed once with T cell culture medium. ConA-induced T cells were aliquoted into 96-well plates (Corning, Tewksbury, MA, USA) at 5 × 10^6^ cells/well in T cell culture medium at a final concentration of 3 ng/mL of purified chIL-2. ConA-induced T cells without added chIL-2 were used as a control group. To investigate the stimulation function of monomeric and dimeric chIL-2 in vivo, each of 4-week old SPF chickens was injected with purified chIL-2 (100 µg i.v. and 100 µg i.p.) or a same volume of PBS (chIL-2 protein solvent) as control [19]. After 72 h of injection, the splenic T cells were isolated. The isolated T cells from in vivo or in vitro experiments were then utilized to evaluate the proportion of CD4^+^ or CD25^+^ T cells among the totally counted cells or T cells (CD3 was used as T cell marker.) by flow cytometry with the following antibodies: PE-conjugated anti-chicken CD3 antibody, FITC-conjugated anti-chicken CD4 antibody (Southern Biotech, Birmingham, AL, USA), and Alexa Fluor 647 (AF647) conjugated anti-chicken CD25 antibody (Bio-Rad Labs, Inc. Hercules, CA, USA). To characterized the binding between monomeric or dimeric chIL-2 proteins and chIL-2 receptor (chIL-2R) in vivo, T cells were isolated within an hour after chIL-2 injection. Hexa-His tagged chIL-2 on purified T cells were detected by Alexa Fluor 647 (AF647) conjugated anti-hexa-His antibody, and analysed by flow cytometry. One-way ANOVA test was used to analyse the significance of differences in stimulatory activity between monomeric and dimeric chIL2 as well as between chIL-2 treated and untreated groups that stimulated with the same source of chIL-2 protein. The bar graphs were plotted using the data that generated from three independent experiments (*n* = 3). Hexa-His tagged chIL-2 proteins that bound to T cells were purified on Ni-NTA Agarose column (Thermo Fisher Scientific Co.). The purified chIL-2 and the CD25 bound to chIL-2 were detected by western blotting using anti-chIL-2 and anti-CD25 antibodies (Bio-Rad Labs).

## Figures and Tables

**Table 1:** The mutants with various combinations of Cys mutation(s).

Single Mutation		Two Mutations	Three Mutations		Four Mutations	
M1 (C41S)		M5 (C41,48S)	M11 (C41,48,94S)		M15 (C41,48,94,97S)	
M2 (C48S)		M6 (C41,94S)	M12 (C41,48,97S)			
M3 (C94S)		M7 (C41,97S)	M13 (C41,94,97S)			
M4 (C97S)		M8 (C48,94S)	M14 (C48,94,97S)			
		M9 (C48,97S)				
		M10 (C94,97S)				

The letters in parentheses indicate amino acids, and the numbers indicate amino acid positions.

**Table 2:** Primers for construction.

Primer Name	Primer Sequence
P1	5′-GTCGACGTCCATGGGAGCATCTCTATCATCAGAA-3′
P2	5′-GCGGCTGCGAAGCTTTTTTTGCAGATATCTCACA-3′
P3	5′-CTCCACAGGTCGACATGTCGTACTACCATCAC-3′
P4	5′-CGCGGTGCGGCCGCTTATCAGTGGTGGTGGTGGTGG-3′
P5	5′-CCGCCGCCGGAATTCATGTCCCCTATACTAGGTTA-3′
P6	5′-GGCCAAGACGTCGACGGATCCACGCGGAACCAGATCCGATTTTGGAGGATGGTC-3′
P7	5′-CACGCACGCGTCGACGATGATGACGATAAAGCATCTCTATCATCAGAAAAATGG-3′
P8	5′-CAGTCTCCCAAGCTTTTATTTTTGCAGATATCTCACAAAGTTGGTCAGTTCATGGAG-3′

The underlined sequences are the restriction sites NcoI in primer P1, HindIII in P2 and P8, SalI in P3, P6 and P7, NotI in P4 or EcoRI in P5, respectively.

**Table 3:** PCR primers for chIL-2 gene mutation.

Primer Name	Primer Sequence
P1(C41S)	5′-GAGACCCAGGAGaGCACCCAGCAAACT-3′
P2(C41S)	5′-AGTTTGCTGGGTGCtCTCCTGGGTCTC-3′
P1(C48S)	5′-GCAAACTCTGCAGTcTTACCTGGGAG-3′
P2(C48S)	5′-CTCCCAGGTAAgACTGCAGAGTTTGC-3′
P1(C94S)	5′-CACCGGAAGTGAAaGCAAGATCTGTG-3′
P2(C94S)	5′-CACAGATCTTGCtTTCACTTCCGGTG-3′
P1(C97S)	5′-GAATGCAAGATCTcTGAAGCTAACAAC-3′
P2(C97S)	5′-GTTGTTAGCTTCAgAGATCTTGCATTC-3′

P1: forward primer; P2: reverse primer. The letters in parentheses indicate amino acids, and the numbers indicate amino acid positions. Bold lowercase letters in sequences are the mutated nucleotides.

## Supplementary Materials

Supplementary materials can be found at https://www.mdpi.com/1422-0067/20/22/5744/s1. Figure S1. Prediction of chIL-2 structure; Figure S2. Alignment of mammalian IL-2; Figure S3. Alignment of human and chicken IL-2; Table S1. The accession numbers of bird IL-2s in alignment; Table S2. The accession numbers of mammalian IL-2s in alignment.

## Abbreviations

IL-2	Interleukin-2
KSCN	Potassium thiocyanate
EDTA	Ethylene Diamine Tetraacetic Acid
β-ME	β-Methylphenethylamine
DTT	Dithiothreitol
Cys	Cysteine
Sf9	*Spodoptera frugiperda*
*E. coli*	*Escherichia coli*
ConA	Concanavalin A
PHA	Phytohemagglutinin
FBS	Fetal bovine serum
EK	Enterokinase
MS	Mass spectrometry
HMS	Honeybee melittin signalling sequence
i.v.	Intravenous injection
i.p.	Intraperitoneal injection
