# Supplementary material for: Four Cysteine Residues Contribute to Homodimerization of Chicken Interleukin-2
Source: Int J Mol Sci. 2019 Nov 15;20(22):5744. doi: 10.3390/ijms20225744 (PMC6888268; doi:10.3390/ijms20225744)
Supplement: Supplementary file 1 [file ijms-20-05744-s001.zip › ijms-604378-prood done supplementary/Supplementary data Legends/Supplementary data Legends.docx]

**Supplementary data Legends of ‘Four Cysteine Residues Contribute to Homodimerization of Chicken Interleukin-2’**

**Figure S1. Prediction of chIL-2 structure. (A)** The model structure of chIL-2 using SWISS-MODEL. **(B)** The crystal structure of human IL-2 (residues 6-133) (PDB#1M47). The C-terminus is in red and the N-terminus is in blue. The conserved Cys residues are coloured in yellow in structure graphs.

**Figure S2. Alignment of mammalian IL-2.** The sequences in rectangles are the signal peptide sequences. The residues highlighted in grey and labelled by asterisk (*) are the conserved Cys residues. Dot indicates no amino acid.

**Figure S3. Alignment of human and chicken IL-2.** The residues highlighted in grey are identical residues. Dot indicates no amino acid.

**Table S1.** The accession numbers of bird IL-2s in alignment.

**Table S2.** The accession numbers of mammalian IL-2s in alignment.
