# Supplementary figures and images for: Four Cysteine Residues Contribute to Homodimerization of Chicken Interleukin-2
Source: Int J Mol Sci. 2019 Nov 15;20(22):5744. doi: 10.3390/ijms20225744 (PMC6888268; doi:10.3390/ijms20225744)

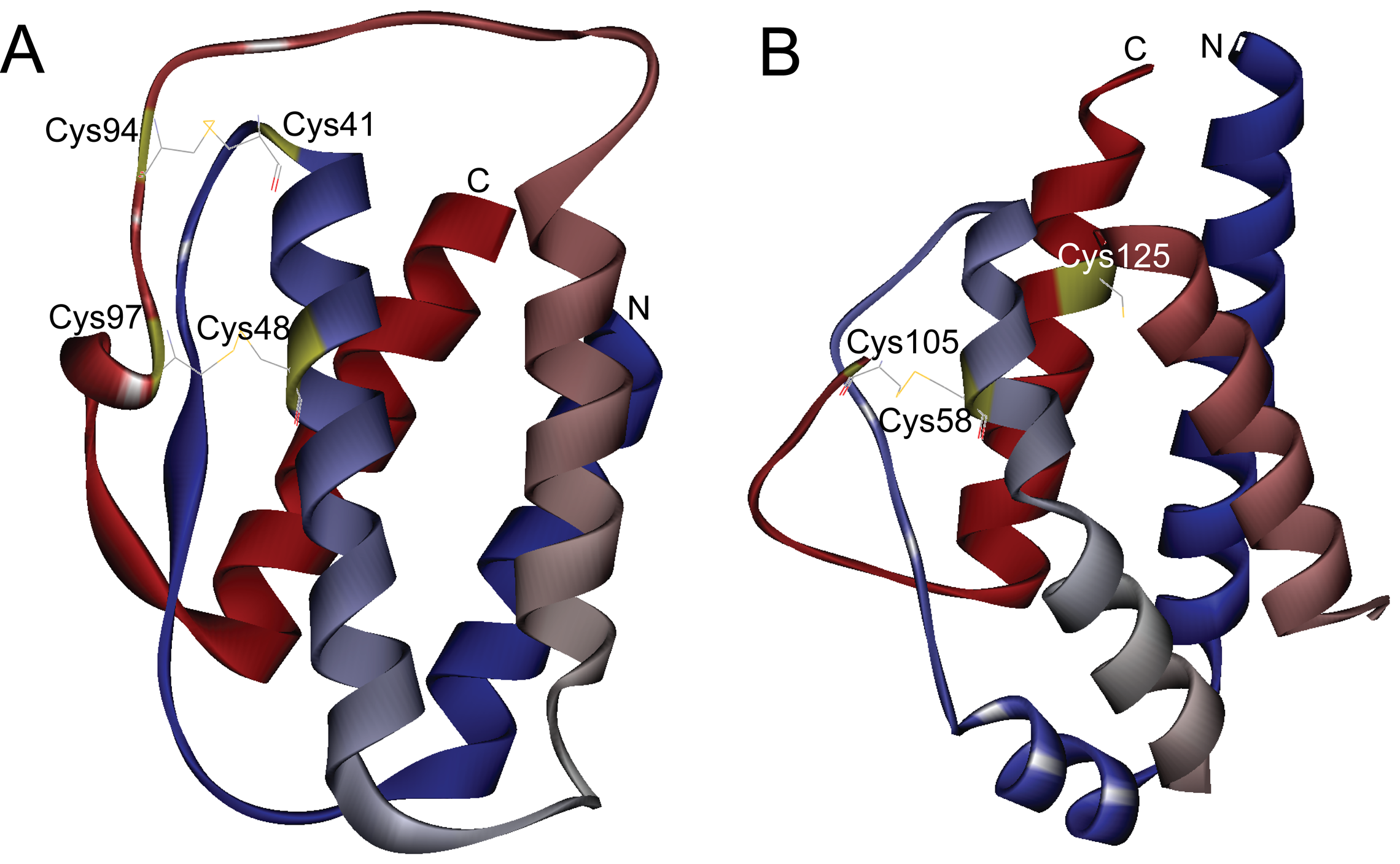

Supplement: Supplementary file 1 [file ijms-20-05744-s001.zip › ijms-604378-prood done supplementary/Supplementary data Legends/Figure S1.tif]

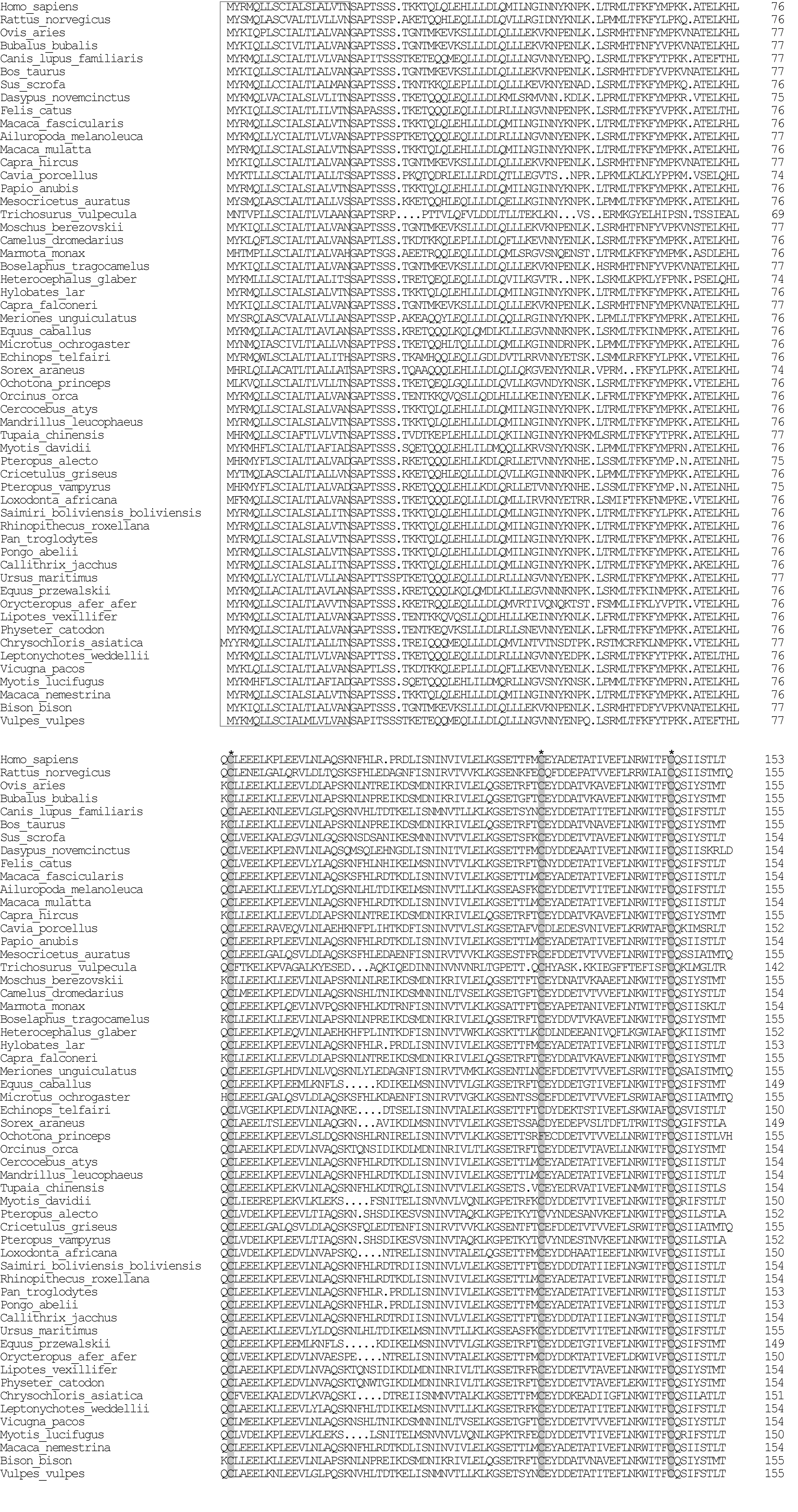

Supplement: Supplementary file 1 [file ijms-20-05744-s001.zip › ijms-604378-prood done supplementary/Supplementary data Legends/Figure S2.tif]

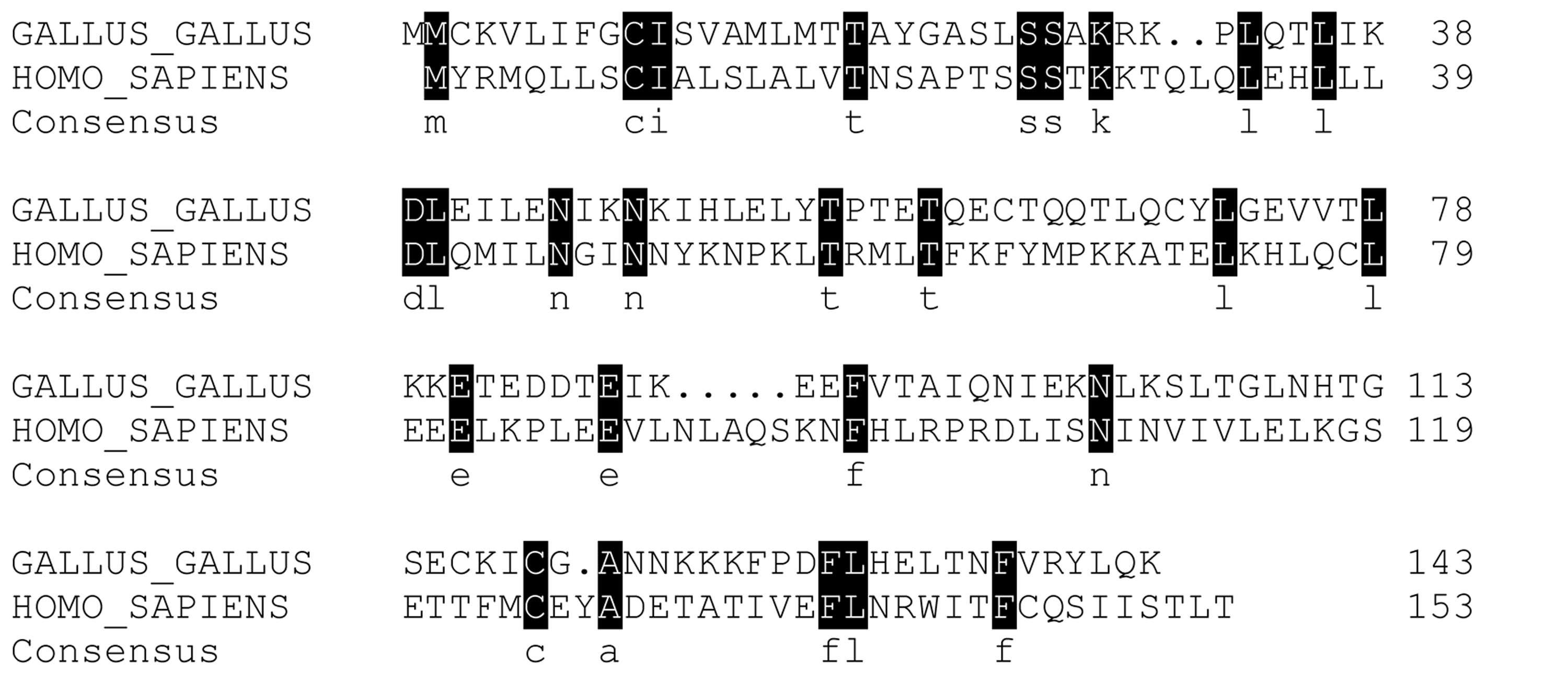

Supplement: Supplementary file 1 [file ijms-20-05744-s001.zip › ijms-604378-prood done supplementary/Supplementary data Legends/Figure S3.tif]
